# Off-Training Levels of Physical Activity and Sedentary Behavior in Young Athletes: Preliminary Results during a Typical Week

**DOI:** 10.3390/sports6040141

**Published:** 2018-11-06

**Authors:** Juliana Exel, Nuno Mateus, Bruno Travassos, Bruno Gonçalves, Isabel Gomes, Nuno Leite, Jaime Sampaio

**Affiliations:** 1 Research Centre in Sports Sciences, Health Sciences and Human Development, CIDESD, CreativeLab Research Community, University of Trás-os-Montes e Alto Douro, UTAD, Quinta de Prados, 5001-801 Vila Real, Portugal; nuno_mateus23@hotmail.com (N.M.); air.bruno.23@gmail.com (B.G.); igomes@utad.pt (I.G.); nleite@utad.pt (N.L.); ajaime@utad.pt (J.S.); 2 Research Center in Sports Sciences, Health Sciences and Human Development, CIDESD, Sport Sciences Department, University of Beira Interior, 6201-001 Covilhã, Portugal; bfrt@ubi.pt

**Keywords:** sports performance, physical activity, accelerometry

## Abstract

The level of physical activity (PA) and sedentary behavior (SED) off-training of young athletes may reveal the quality of recovery from training and highlight health related issues. Thus, the aim was to identify and describe young athletes’ PA and SED off-training, according to daily life activities. Eight athletes (15.7 ± 2 years, 1.72 ± 0.6 m height, 62.9 ± 10.2 kg) of a sport talent program wore on their waist a tri-axial accelerometer (ActiGraph^®^ wGT9X-link, Shalimar, FL, USA) at 30 Hz for 15 consecutive days, and reported their schedule. A two-step cluster analysis classified three groups according to sedentary PA and MVPA. The Sedentary (56.9%), presented the highest sedentary PA (mean [CI], 37.37 [36.45–38.29] min/hour); The Hazardous (19.4%) had the lowest values of sedentary and MVPA (10.07 [9.41–10.36] min/hour and 8.67 [7.64–9.70] min/hour, respectively). Balanced (23.7%) had the highest MVPA (28.61 [27.16–30.07] min/hour). Sedentary had the lowest count of home time associated (20%) and higher school (26%) time when compared to the Hazardous (13%). The Balanced showed the highest count of school (61%) and home time (47%). Different profiles for young athletes revealed alarming behavior in the associations with sedentary PA, sitting and SED breaks, which may influence performance and health.

## 1. Introduction

Physical activity (PA) and sedentary behavior (SED) profiles of healthy [1,2,3] and clinic-conditioned youth [4,5], considering weekly variability, its influence on activity patterns [6,7], and socio-economic profiles [8], have recently pointed out that modern life is contributing to increased sedentariness and also its hazardous outcomes. Accordingly, the World Health Organization (WHO) recommends children and adolescents to accumulate at least 60 min of moderate-to-vigorous physical activity (MVPA) daily to generate cardiovascular, neuromuscular and metabolic benefits [9].

Previous studies demonstrated independency between time spent in MVPA and SED by evidencing that people who accomplish the recommended MVPA levels, when compared to sedentary ones, do not present less SED time [10,11]. However, the time spent in MVPA and in sedentary PA impact health status. For instance, it is clear that the time spent in MVPA is associated with physical fitness of youths [1], while the time of SED can be associated with the risk of cardio metabolic diseases [12]. There is evidence of decline in the levels of habitual physical activity with an associated decline in maximal aerobic performance in children and adolescents through time [13], which makes the promotion of youth health and well-being challenging. The different environments involved in daily-life activities may be a constraint affecting the different PA and SED settings for children and adolescents. Studies have found that PA in school environment is lower than out of school for children, especially at secondary school ages [14]. After-school periods has also been reported as presenting high screen and non-screen SED among adolescents [15].

To young athletes, in the perspective of sports performance, PA and SED profiles should also be considered key to evaluating, preventing and treating overtraining symptoms [16]. Indeed, the level of PA and SED off-training may reveal not only information regarding the quality of rest and recovery from training hours, but also highlight issues related with health status parameters of young athletes. Although young athletes may perform MVPA beyond the recommended level due to training and competitions, it is suggested that they also be active and maintain healthy habits during off-training hours. In addition, young athletes experience stress as well as habits of sedentary behavior regarding ordinary daily life activities and social behaviors developed between youth. Therefore, despite the MVPA achieved during the training and competitive time, young athletes might not be excused from the alarming consequences of sedentariness.

Thus far, two studies have analyzed the PA and SED profiles during off-training hours and it seems that elite athletes have higher levels of SED during waking hours when compared to non-athletes [17,18]. This evidence raised questions over the management of off-training time for optimal recovering and the maintenance of positive health status. It is difficult for athletes, coaches, and trainers to select choices about the best recovery strategy among all sports modalities available, however, individualization of the needs and context-based approach in daily-life schedule structure seems to be critical [19].

The efforts for measuring, interpreting and managing internal and external training loads in sports [20] or even in the practice of physical activity are massive [21]. Currently, athletes of different competitive levels and people that practice physical activity have embraced the use of gadgets and electronic devices for the management of internal and external training loads during practice. It facilitates monitoring of training [22] and recovering loads in athletes [23], the assessment of sleep quality [24] and the impacts of physical activity on health indicators in diverse groups of people [25,26]. There is also an important contribution to maintaining young athletes engaged in physical activities, thus guaranteeing longevity to their careers [27]. In addition, the online feedback and constant rewards and encouragement provided by wearable technology helps on the identification of progress and health status. Also, social competition and community participation [28] are some of the benefits for using wearable technology between athletes of different competitive levels.

In line with that, wearable accelerometers have been extensively used as a non-invasive way to estimate energy expenditure levels in both competitive and free-living environments [1,11,17,18,29,30,31]. However, there are still gaps on literature about how to interpret this sort of data and provide meaningful insights to improve the quality of interventions, considering the multidimensionality of stress-recovery state of individuals [32]. Thus, the aim of this study was to identify and describe young athletes’ level of PA and SED off-training and according to their usual activities schedule, using wearable accelerometry technology. It was hypothesized that the outcome variables can classify and reveal vital information on the levels of PA and SED in young athletes, especially regarding the activity patterns during typical week days.

## 2. Materials and Methods

### 2.1. Participants

Eight young athletes (15.7 ± 2 years, 1.72 ± 0.6 m height and 62.9 ± 10.2 kg) of a high-level sports talent program were recruited to participate in this study. Criteria for inclusion were applied to ensure all athletes were aged 13–17 years, were engaged in a minimum of six hours of training per week (with additional competition once a week), and had professional experience of no less than five years of regular participation at previous competitive seasons. Athletes, their legal guardians, and coaches were fully informed about the purpose, benefits and risks of the study, and provided written informed consent before the study started. The study protocol conformed to the recommendations of the Declaration of Helsinki. It was approved and followed the guidelines stated by the local Institutional Research Ethics Committee.

### 2.2. Protocol

The protocol design consisted of two evaluation moments. In the first moment, the athletes received the Actigraph^®^ GT9X Link + (Pensacola, FL, USA) to monitor their PA and SED, as well as the instructions of how to use it. They should wear the monitor in the dominant side of their hip, for 15 consecutive days, completing valid mean wear time of 10.6 ± 1.0 h per day. They were asked to not use the devices during training periods, bed time, personal care and activities under water. Participants were asked to fill a daily report with a rough description of the periods of monitor wearing and non-wearing. The participants also described in this report their schedule of activities performed during the day, according to the following categories: school, home time, leisure time, training, and competition. The second evaluation moment was arranged at the end of accelerometer data recording period to collect the monitors and the daily report.

### 2.3. Measures

As a valid instrument to measure PA and SED in free-living environment [33], the triaxial acceleration recorded by the monitoring device at 30 Hz of sampling frequency was processed using ActiLife^®^ 6.10 software (ActiGraph LLC, Fort Walton Beach, FL, USA). The data were chunked in 60-s epochs. Sixty or more minutes of zero accelerometer counts were considered non-wear time, therefore excluded from analysis. Valid accelerometer wear time consisted of, at least, 600 min of accelerometer wear time per day [34] and a minimum of eight weekdays of data were considered to represent an athlete’s weekly profile. The energy expenditure algorithm used was the Freedson Combination [35]. The cut points used to calculate the variables associated to each PA intensity was based on Romanzini et al. for children and adolescents [36]: sedentary PA was considered 0–180 counts·min^−1^, light PA was 181–756 counts·min^−1^, moderate PA was 757–1111 counts·min^−1^, and vigorous PA was ≥1112 counts·min^−1^. Sedentary breaks (SED breaks) were as defined as time spent in a minimum of 30 min of prolonged sedentary activity specifically in each waking hour of the day. The sedentary behavior was analyzed through the ActiGraph^®^ inclinometer measures and associated to the following modes: sitting, laying and standing.

### 2.4. Data Analysis

All PA and SED variables were analyzed in minutes per hour during off-training time. The athletes were classified in groups using a two-step cluster analysis according to the hourly distribution of MVPA and sedentary PA, which was considered the main variables in representing PA and SED off-training beforehand. One-way ANOVA was used to test the differences between MVPA and sedentary PA among the groups defined by the cluster model.

Then, how other PA and SED variables (energy expenditure, MET, light PA, moderate PA, vigorous PA, standing, lying, sitting, and step counts) would distinguish the clustered groups was tested, as well as its efficiency in correctly classifying the original groups, but only for the periods where SED breaks were higher than 30 min, using discriminant function analysis. The effect sizes of the discriminant functions were assessed by Wilks’ lambda and squared canonical correlation, which explains the variance associated with each function [37]. Additionally, to determine which examined variables contributed to player group differences, the structure coefficients were evaluated. A cross-tabulation was used to verify the relationship between the clustered groups and activity patterns described by the athletes in their typical week schedule. All the analyses were performed in the software IBM SPSS^®^ (Armonk, NY, USA: IBM Corp.), with *p* < 0.05.

## 3. Results

This study aimed to identify and describe young athletes’ level of PA and SED off-training and according to their usual activities schedule. Thus, the two-step cluster analysis revealed that sedentary PA and total MVPA can be grouped into three different clusters, with a good average silhouette coefficient of 0.6 [38]. Means and confidence intervals of the PA and SED variables totals and per cluster group are described in Table 1. Most data were grouped in Cluster 1 (56.9%), followed by Cluster 3 (23.7%) and Cluster 2 (19.4%). Sedentary PA had higher within group importance for Clusters 1 and 2 than total MVPA. Cluster 1 was assigned as the Sedentary group, once it presented the highest mean for sedentary PA, sitting and lying, and lower moderate PA and SED breaks. Cluster 2 was named as the Hazardous group, for the lowest values of SED and PA variables, except SED breaks, which was the highest among groups. Cluster 3 was the Balanced group, for which MVPA had the highest within cluster importance in the model of separation, and presented the highest means of the PA variables, as well an amount of SED variables and modes. 

**Figure 1:**
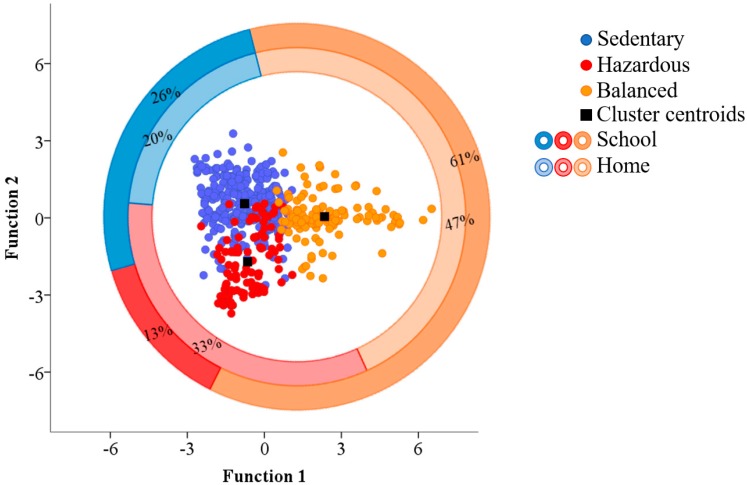
Scatterplot of all-groups and respective centroids of the two discriminant functions defined by the clustering model. The outer and inner grand circles represent the crosstabulation results of the young athletes’ school and home time schedule, respectively, distributed according the groups.

The discriminant analysis revealed that two functions can explain all the variance contained in the dataset. The first and second functions hold 70.4% and 29.6% of the total variance explained, respectively, with statistically significant Wilks’ lambda (0.21 and 0.58 for Functions 1 and 2, respectively, *p* < 0.001). Vigorous PA, steps counts, energy expenditure, standing, moderate PA and MET had the highest correlation with the first function, while lying, SED breaks, sitting and light PA had highest correlation to the second function (Table 1). The scores of the two discriminant functions as function of the cluster groups and the concomitant centroids are shown in Figure 1. A total of 88.7% of original grouped cases were correctly classified with the two discriminant functions obtained. Sedentary group had 94.6% of the cases grouped accordingly, while Hazardous had 69.3% and Balanced had 90.2%. Figure 1 also represents the percentage of the category schedule (home/school) in describing the groups.

## 4. Discussion

The present study characterized the PA and SED of young athletes during non-training hours. Findings revealed three distinct profiles of MVPA and sedentary PA spent on daily basis. The Sedentary group showed the highest mean sedentary PA per hour of waking time, while the Balanced group presented the highest mean MVPA per hour of waking time, and the Hazardous group had the lowest sedentary PA and MVPA. Both Sedentary and Balanced, however, achieve the WHO daily recommended levels of MVPA, considering the mean wear time for the sample of this study was 11 h. Previous studies have reported that PA and SED are distinct domains not only for those who do not meet the recommended guideline levels, but also for those who do, including young people [39] and athletes [10,40].

Thus, present results indicate that the displacement among these behaviors is also present in young athletes’ lifestyle. Indeed, the levels of sedentary PA off-training found for all groups has already been reported for elite professional soccer players at post-training time as well [17]. Non-training hours for young athletes are diving among academic activities, leisure, and rest. However, successful training programs should be able to balance the application of loads needed by an athlete to achieve peak performance, avoiding the negative consequences of overtraining [41] through the application of high quality recovery. The path to find the sweet spot between quality recovery without the negative outcomes of sedentariness may be on the assumption of SED as a complex behavior. Consequently, it should be studied and explored in its various manifestations and accounted for the respective environments and activities involved according to age [42]. SED can be related to certain postures or movement patterns as standing, lying and sitting, which demand low energy expenditure [43]. It can also be related to the environment where these low energy movements are performed. Typical work or school-related SED include sitting at a desk and leisure-time behaviors involve home screen-related activities such as TV viewing, cell phone interaction or recreational computer use. Thus, the profiles obtained for all three groups found by the clustering model applied to the data interestingly characterized how SED and PA is distributed in the different environments reported by the young athletes in their daily schedule report. The Sedentary group profile is highlighted by the daily time spent in moderate PA and SED breaks, sitting and lying, which were the variables highly correlated to the second discriminant function, which was highly effective in classifying this group. Sedentary group showed less daily SED breaks when compared to the other groups, but lower daily time in moderate PA and higher time in sitting mode. Higher amounts of daily total sitting time is associated with all-cause mortality risk in adults [44]. The crosstabulation of this data with the daily schedule revealed that this group has the lowest count percentage of home time associated, but higher school time when compared to the Hazardous group. Thus, the time at school for the young athletes in the Sedentary favors alarming sedentary behavior. Literature indicates that the levels of moderate intensity PA can attenuate, but not eliminate that risk of all-cause mortality [45] in adults, although may improve markers of cardiometabolic risk in young people [46]. The focus on decreasing sitting time and increasing moderate PA could benefit the athletes classified in this group.

The Balanced group was characterized by higher MVPA, light, moderate and vigorous PA, as well as step counts. The first discriminant function, which explains most of the data variability, shows high and significant correlations to these variables. This group did not present the lowest daily time in sedentary PA; hence, off-training, it seems that the athletes classified in this group better manage their PA and SED profiles throughout the day. The Balanced group showed the highest count percentages of school and home time, indicating that their profiles are not affected by the main environments attended in daily life, thus there is viability in finding balance between PA and SED off-training in modern daily life for athletes. This could be used as parameter of reference for outcomes in the planning of programs and activities for the ones classified in the other groups.

This is not the case for the Hazardous group, which showed the lowest mean daily time spent in all PA and SED variables. This result did not avoid the number of SED breaks, however, being the highest among the groups. Prolonged sedentariness in children and adolescents is associated with increased body fat in athletes [1,11], obesity [42] and its persistence in childhood [4], poor cardiorespiratory fitness, lower insulin sensitivity, higher blood pressure and total cholesterol, and compromised academic achievement [3,42,47]. School time had the lowest count percentage for the Hazardous group, hence most of their PA and SED profile comes from considerable time at home. Home time can be related to rest and recovery for children and adolescents. Extended sleep additional to a day off from training is a recommended strategy for physical recovery [48], as well as for general well-being. However, light and MVPA has been reported to be more effective than passive rest as recovering strategy [49].

It might be argued that young elite athletes being exposed to hours of training and competitions is enough to address this issue while enhancing fitness components [50] and decreasing injuries by overuse [51] and adequate application of training loads. Organized sport should be encouraged as an efficient way to promote higher levels of PA in youth [52] and increase fitness level [53], but is often underestimated [54]. Consequently, it also might be argued that, because recovery is essential to athletic performance, sedentary time of young athletes is not an issue. The line between the efficacy of training, recovery and healthy behavior is tenue and dependent on the environment where the athletes spend most of their time, as emphasized by our results.

Age- and gender-related patterns influence time use in terms of PA and SED [55], but participation in high-level sport is also a social and biological factor that changes how these relationships are interpreted in young people. The selected subjects for this study are very talented athletes and were chosen as the best among their peers. With limitations in terms of sample size, which is common when studying high-level athletes, it was possible to identify that PA and SED data during non-training hours were highly discriminatory. In addition, crossing these profiles with the schedule report results, it was possible to identify and contextualize athletes’ PA and SED profiles across their main daily life activities. Thus, to apply and meaningfully interpret the outcomes of the monitorization of physical and physiological condition outside elite sport environment may reveal important information to help trainers and coaches controlling whether athletes´ activity behavior meets the performance aims and health guidelines, towards a successful and healthy development. Future studies can advance in associating data on athletes of different sport practice context to risk levels in terms of health and performance.

## 5. Conclusions

The aim of this study was to identify and describe young athletes’ level of PA and SED off-training and according to their usual activities schedule in a typical week. Different patterns were found for young athletes’ PA and SED profiles, revealing occurrence of alarming behavior in the time spent in sedentary PA, sitting and prolonged sedentary breaks during the day. Most weekdays waking hours are spent at school or home, which are places that promote sedentarism. However, some athletes still manage to balance healthy PA and SED levels. The awareness of such profiles is important for performance, which might help in the recovering process of training, as well as for health, once it decreases the harmful influences of the inevitable modern sedentarism. Thus, this information may provide useful data and insights for helping coaches and trainers in controlling the loads that may influence training performance as well as health status.

## Figures and Tables

**Table 1:** Group and totals daily means [confidence intervals] per hour for the PA and SED variables of young athletes off-training during a typical week.

Variables	Sedentary	Hazardous	Balanced	Totals	Function 1	Function 2
Energy expenditure (kcals)	22.52 [20.52–24.53]	15.11 [12.94–17.28]	73.48 [65.96–81.00]	29.66 [27.33–31.99]	0.69 *	0.17
MET	1.06 [1.05–1.07]	1.09 [1.06–1.12]	1.22 [1.18–1.27]	1.10 [1.09–1.11]	0.32 *	−0.07
Sedentary PA (min)	37.37 [36.45–38.29]	12.63 [11.12–14.14]	17.20 [15.86–18.55]	32.02 [30.93–33.10]	n/a	n/a
Light PA (min)	11.43 [10.80–12.06]	9.21 [7.22–11.19]	13.06 [12.02–14.09]	9.78 [9.31–10.26]	0.10	0.16 *
Moderate PA (min)	2.25 [2.03–2.46]	2.31 [1.88–2.74]	4.64 [4.15–5.13]	2.46 [2.29–2.62]	0.36 *	0.01
Vigorous PA (min)	7.82 [7.23–8.41]	6.36 [5.53–7.19]	23.98 [22.46–25.49]	9.99 [9.36–10.63]	0.90 *	0.16
Total MVPA (min)	10.07 [9.41–10.73]	8.67 [7.64–9.70]	28.61 [27.16–30.07]	12.45 [11.75–13.16]	n/a	n/a
Step counts	303.06 [279.05–327.06]	210.34 [178.46–242.22]	912.80 [843.90–981.69]	389.00 [360.65–417.35]	0.80 *	0.20
Standing (min)	10.33 [9.56–11.10]	8.26 [6.98–9.55]	25.00 [22.95–27.04]	11.73 [11.03–12.44]	0.61 *	0.15
Sitting (min)	5.24 [4.19–6.28]	1.83 [1.06–2.60]	2.00 [1.22–2.79]	5.14 [4.45–5.84]	−0.11	0.20 *
Lying (min)	37.70 [35.81–39.60]	17.87 [14.61–21.13]	25.59 [23.36–27.82]	31.20 [29.88–32.51]	−0.16	0.56 *
Sedentary breaks over 30 min (min)	47.18 [46.03–48.32]	57.24 [56.09–58.38]	56.57 [55.35–57.78]	51.37 [50.52–52.20]	0.27	−0.51 *

Total MVPA, total moderate-to-vigorous physical activity; SED breaks, time spent in at least 30 min of prolonged sedentary activity; MET, metabolic equivalent; *, largest absolute correlations associated with each discriminant function; n/a, non-applicable.
